# Relationship of early-life trauma, war-related trauma, personality traits, and PTSD symptom severity: a retrospective study on female civilian victims of war

**DOI:** 10.3402/ejpt.v7.30964

**Published:** 2016-04-06

**Authors:** Aleksandra Stevanović, Tanja Frančišković, Eric Vermetten

**Affiliations:** 1 Department of Psychiatry and Psychological Medicine, School of Medicine, University of Rijeka, Rijeka, Croatia; 2 Military Mental Health Research, Department of Defence, Utrecht, The Netherlands; 3 Department of Psychiatry, Leiden University Medical Center, Leiden, The Netherlands; 4 Arq Psychotrauma Research Group, Diemen, The Netherlands

**Keywords:** War, PTSD, civilian victims of war, female, personality traits

## Abstract

**Background:**

Consequences of war-related traumatisation have mostly been investigated in military and predominant male populations, while research on female civilian victims of war has been neglected. Furthermore, research of post-war posttraumatic stress disorder (PTSD) in women has rarely included early-life trauma in their prediction models, so the contribution of trauma in childhood and early youth is still unexplored.

**Objective:**

To examine the relationship of early-life trauma, war-related trauma, personality traits, and symptoms of posttraumatic stress among female civilian victims of the recent war in Croatia.

**Method:**

The cross-sectional study included 394 participants, 293 war-traumatised adult women civilians, and 101 women without war-related trauma. Participants were recruited using the snowball sampling method. The applied instruments included the Clinician-Administrated PTSD Scale (CAPS), the NEO Personality Inventory-Revised (NEO-PI-R), the War Stressors Assessment Questionnaire (WSAQ), and the Early Trauma Inventory Self Report-Short Form (ETISR-SF). A hierarchical multiple regression analysis was performed to assess the prediction model of PTSD symptom severity measured by CAPS score for current PTSD.

**Results:**

The prevalence of current PTSD (CAPS cut-off score=65) in this cohort was 20.7%. The regression model that included age, early-life trauma, war-related trauma, neuroticism, and extraversion as statistically significant predictors explained 45.8% of variance in PTSD symptoms.

**Conclusions:**

Older age, exposure to early-life trauma, exposure to war-related traumatic events, high neuroticism, and low extraversion are independent factors associated with higher level of PTSD symptoms among women civilian victims of war.

The number of civilian victims of wars has been rising since the 1990s with some reports stating that up to 90% of all war victims are non-military (Council of the European Union, 2009). However, factors associated with the development of posttraumatic stress disorder (PTSD) after experiencing war-related trauma have mostly been investigated in military and dominantly male populations (Brewin, Andrews, & Valentine, 2000; Ozer, Best, Lipsey, & Weiss, 2003). Prevalence rates of PTSD among community samples affected by war vary significantly from 11% in students following the air-attacks (Gavrilovic et al., 2003), 16–37% among civilians in Algeria, Cambodia, Ethiopia and Gaza (De Jong, Komproe, & Van Ommeren, 2003), yet even up to 61% among young people in Baghdad (Al-Hadethe, Hunt, Thomas, & Al-Qaysi, 2014). In a methodologically sound study on the prevalence of mental disorders after the war in the Balkans in adult population still living in the area of conflict, the prevalence rates of PTSD were 10.6% in FYR Macedonia, 18.0% in Croatia, 18.2% in Kosovo, 18.8% in Serbia, and 35.4% in Bosnia and Herzegovina (Priebe et al., 2010). Studies on civilian victims of war have established that female gender, older age, and cumulative effect of war-related trauma are risk factors for developing PTSD (Johnson & Thompson, 2008; Priebe et al., 2010).

A neglected factor associated with the development of posttraumatic symptoms after the war is exposure to early, pre-war trauma, especially in childhood and early youth years (Cloitre et al., 2009; Owens et al., 2009). Early studies have reported higher rates of childhood trauma in combat veterans with PTSD as compared with soldiers who have not developed the disorder (Bremner, Southwick, Johnson, Yehuda, & Charney, 1993; Clancy et al., 2006). A study conducted in a representative sample in the general population has found that previous exposure to trauma, either in childhood or later in life, was associated with a higher risk of PTSD from trauma in adulthood (Breslau, Chilcoat, Kessler, & Davis, 1999). It has also been proposed that an increasing number of different types of traumas was associated with an increasingly greater number of different types of symptoms experienced simultaneously (Briere, Kaltman, & Green, 2008; Van der Kolk, Roth, Pelcovitz, Sunday, & Spinazzola, 2005). Briere et al. (2008) tested this notion and found a linear relationship between the number of trauma types experienced before the age of 18 and symptom complexity in a community sample of young women (college students).

In addition to the aforementioned risk factors, it has also been shown that personality traits play an important role in the development of PTSD (Bramsen, Dirkzwager, & Van der Ploeg, 2000; Jakšić, Brajković, Ivezić, Topić, & Jakovljević, 2012; Ozer et al., 2003). Personality traits are defined as patterns of behaviour, thoughts, and emotions that remain stable over time. Even though a scientific consensus on basic personality traits has not been reached, commonly used concept is that of the five-factor model that includes neuroticism, extraversion, openness, conscientiousness, and agreeableness (Jakšić et al., 2012).

There is robust evidence of a correlation between neuroticism and PTSD. Studies on war veterans report a significant relationship between PTSD and the level of neuroticism before and after trauma exposure (Bramsen, Van der Ploeg, Leo, & Adèr, 2002; Cox, MacPherson, Enns, & McWilliams, 2004; Engelhard & Van den Hout, 2007). The relationship has been confirmed in burn victims (Lawrence & Fauerbach, 2003) and elderly people with myocardial infarction (Chung, Berger, Jones, & Rudd, 2006). The results concerning the other four personality traits and their relationship with PTSD are still inconsistent. According to a longitudinal research on self-reported, adverse life events and longitudinal changes in the five-factor model personality traits, conducted on an urban sample, the following are the predictors of poor mental health after exposure to trauma: a lower score on extraversion and/or conscientiousness at baseline, increases in neuroticism, and a reported extreme event (Löckenhoff, Terracciano, Patriciu, Eaton, & Costa, 2009; Specht, Egloff, & Schmukle, 2011). In addition, after experiencing extremely stressful event(s), there is a significant decrease on openness and agreeableness compared to pre-trauma measure (Löckenhoff et al., 2009). Elder people with myocardial infarction and PTSD, beside higher score on neuroticism, scored lower on agreeableness compared with participants without PTSD (Chung et al., 2006). Results from a longitudinal study show that neuroticism is the strongest predictor of PTSD in elder people, followed by lower conscientiousness and lower agreeableness (Ogle, Rubin, & Siegler, 2014). Karanci et al. (2012) have investigated the effects of personality traits and posttraumatic symptoms on posttraumatic growth in a community sample. The results showed that conscientiousness, agreeableness, and openness to experience are significantly related to the posttraumatic growth, while the effects of extraversion, neuroticism, and openness to experience were moderated by the severity of posttraumatic symptoms for some domains of posttraumatic growth.

An extensive body of literature shows that women develop PTSD more often than men (Christiansen & Hansen, 2015; Chung & Breslau, 2008; Tolin & Foa, 2006), although men generally report a more frequent exposure to traumatic experiences (Kessler, Sonnega, Bromet, Hughes, & Nelson, 1995; Tolin & Foa, 2006). The lifetime prevalence of PTSD is 10.4% for females and 5.0% for males and the conditional risk across trauma types is 20.4% for females and 8.2% for males (Kessler et al., 1995). In the context of armed conflict, women in civilian populations may be exposed to the same traumatic experiences as active soldiers (bombarding, missiles, being surrounded by the enemy, etc.), yet women may be additionally exposed to a wide range of specific gender-based violent acts, such as forced pregnancy, abduction, rape, sexual slavery, and forced prostitution during wars (Hynes, 2004). War in Croatia took place between 1991 and 1995. Fifty-four per cent of the state territory inhabited by 36% of the population was under direct war-related activities. Civilian population was severely affected. It is estimated that the number of civilians killed or died because of wounding is between 4,000 and 8,000, with more than 550,000 displaced persons (Bužinkić, 2012). In addition, the post-war context in which most of the civilians lived included social, economic, and political instabilities.

Although in the last two decades, factors like personality traits and early trauma in PTSD development have gained some attention, we believe there is a lack of research done on civilians, especially women, who have experienced prolonged war-related traumatisation. The present study examines the relationship between early trauma, exposure to war-related trauma, personality traits, and severity of PTSD symptoms among female civilian victims 10 years after the war in Croatia.

Based on previous research, it was hypothesised that a higher exposure to early trauma in combinations with a higher number of war-related traumatic events would be predictors of posttraumatic stress symptoms. It was also hypothesised that personality traits from the five-factor model, especially higher neuroticism and lower extraversion, conscientiousness, agreeableness, and openness would explain additional variance in posttraumatic symptomatology among the traumatised civilian women.

## Methods

Data presented in this study are drawn from the Project Psychobiology of PTSD (PBPTSD) carried out through the Sixth Framework Programme, Priority INCO—Western Balkan countries in Croatia, Serbia, Italy, and the Netherlands, from October 2004 to July 2008 (www.cordis.europa.eu/project/rcn/75649_en.html).

The project was designed as a comprehensive cross-sectional study encompassing both psychological and biological investigations performed on individuals with PTSD, individuals with cured PTSD, individuals with traumatic experiences without PTSD, and a group of healthy controls. Power analysis and sample size calculation suggested that 92 subjects per group allow us to detect population group differences. Thus, the sample size of 300 participants with war-related trauma was set in order to assess women with different PTSD status and 100 volunteers who have not experienced war traumatisation.

### Participants

A total of 506 women were contacted using the snowballing method. This is a non-probability sampling technique where existing study subjects recruit future subjects from among their acquaintances. Despite some methodological shortcomings, snowballing method allows access to hidden and difficult-to-reach populations (Faugier & Sargeant, 1997). Initial contacts were made through government and non-government associations formed to assist war victims in several Croatian cities. Each participant that completed assessment was asked for referral of the next possible participant(s). Of the initial 506 possible participants, 417 were included in the further assessment while 89 refused to participate in the study due to lack of time or because they did not want to remember the events related to the war. The general inclusion criteria were participants aged between 18 and 65, whose level of education and degree of understanding satisfy the requirements of adequate communication with investigators and completion of psychological tests. The exclusion criterion was the presence of severe physical illness and/or health condition that might have exposed the respondent to a higher risk for the occurrence of adverse situations or that might have affected the assessment during the study. Other exclusion criteria were as follows: acute psychotic reaction at the time of the assessment, alcohol dependence, or alcohol abuse in 6 months preceding the assessment and/or psycho-organic syndrome. These disorders were diagnosed according to the DSM-IV, Axis 1, and the selection was made through the Structured Clinical Interview SCID-I. None of the participants met the criteria for the acute psychotic reaction, alcohol dependence, alcohol abuse, or psycho-organic syndrome.

**Fig. 1:**
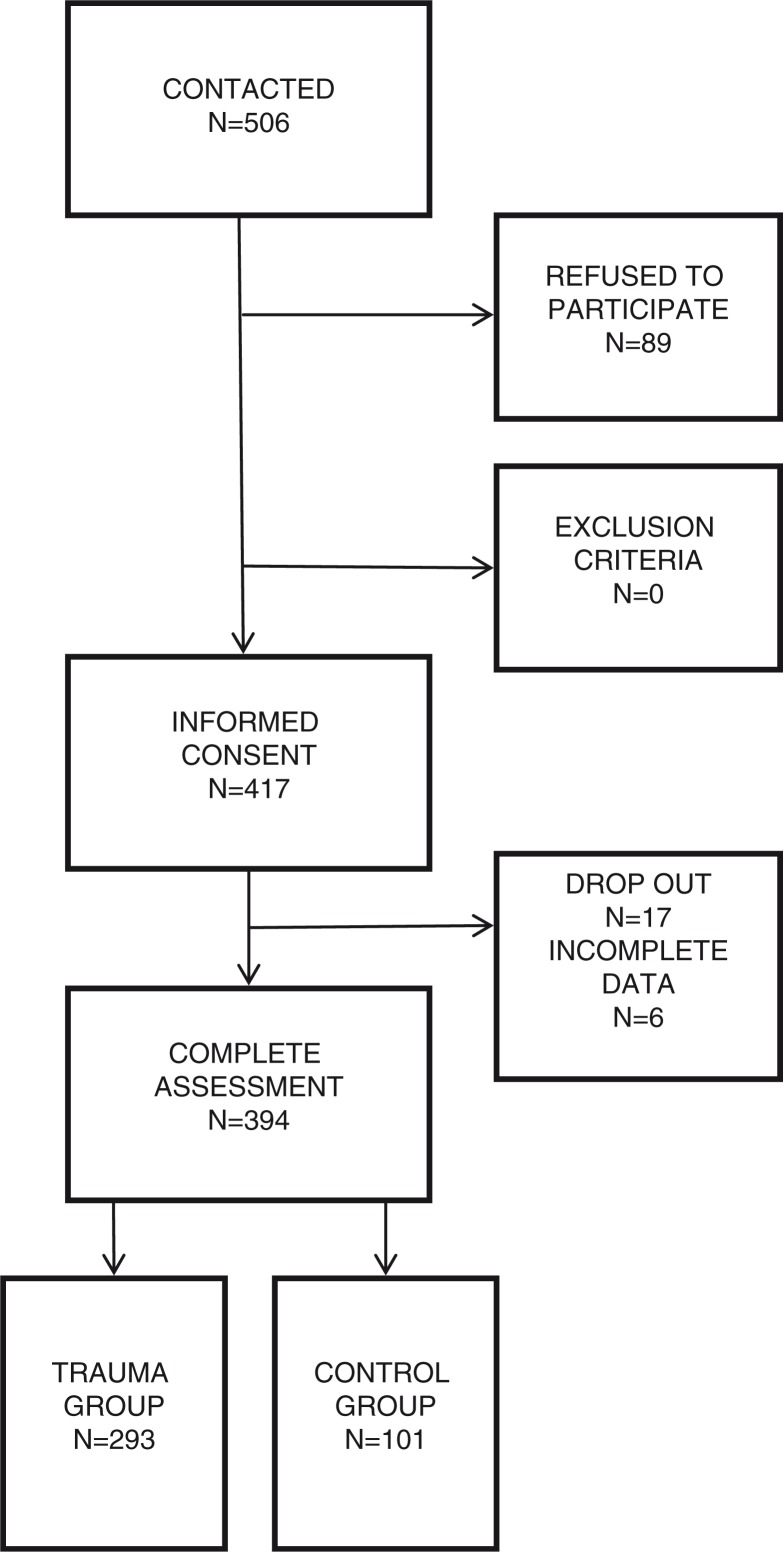
Flowchart of the inclusion of the participants.

None of the participants met the exclusion criteria, but 17 participants dropped out after the initial screening and 6 participants were excluded from further analyses due to a large amount of missing data in self-reporting instruments. The final sample consisted of 394 participants who were further divided into “trauma” or “control” group based on the specific criteria of having experienced at least one war-related traumatic event. The flow diagram of the number of participants included in the study is presented in Fig. 1.

The average age of the participants in the trauma group (*N*=293) was 41.13 (SD=11.71) and ranged from 21 to 65 years. Years of education ranged from 4 to 20 years (*M*=12.20, SD=2.8). The majority of the participants were married or living in cohabitation with a partner (50.9%), single (16.9%), divorced (10.2%), or widowed (21.7%). The average monthly household income was €564.14 (SD=528.58). Participants in the control group (*N*=101) did not differ in age (*M*=42.4, SD=13.32) (*t*=0.814; *p*=0.386), marital status (married/cohabitating 50.5%, single 32.7%, divorced 10.9%, widowed 5.9%) (*χ*^2^=1.046; *p*=0.790) and average monthly household income (M=479.6, SD=363.4) (*t*=1.113; *p*=0.269) compared to trauma group. The only significant difference between groups was observed in years of education (M=13.1, SD=3.51) (*t*=2.378; *p*=0.019). Participants in the control group on average have significantly higher level of education compared to trauma group.

### Instruments

Data on age, educational level, marital status, and income were collected through the socio-demographic questionnaire created for the purposes of this study.

In order to assess the severity of PTSD, we used the Clinician Administrated PTSD Scale (CAPS). The CAPS is a structured interview designed to make a categorical PTSD diagnosis, as well as to provide a measure of PTSD symptom severity. The structure corresponds to the DSM-IV criteria, with B, C, and D symptoms rated for both frequency and intensity; these two scores are summed to provide severity ratings (First, Spitzer, Gibbon, & Williams, 2000). The cut-off score of 65 was employed to determine the prevalence of PTSD. Weathers, Ruscio, and Keane (1999) employed signal detection methods to identify the optimally efficient cut-off score for the prediction of PTSD diagnoses. A CAPS total severity score of 65 had 0.82 sensitivity, 0.91 specificity, 0.86 efficiency and kappa of 0.78 against the SCID PTSD diagnosis.

For the assessment of personality traits, we applied the NEO-Personality Inventory-Revised (NEO-PI-R) (Costa & McCrae, 1992). The NEO-PI-R is a self-report questionnaire that measures five personality dimensions: neuroticism, extraversion, openness, conscientiousness and agreeableness. Raw scores for each dimension were used in the analyses.

In order to examine frequency of war-related traumas, we administered the War Stressors Assessment Questionnaire (WSAQ) (Jović, Opačić, Knežević, Tejnović, & Lečić-Toševski, 2002). The questionnaire was developed in order to cover a wide spectrum of stressors characteristic of war conflict in former Yugoslavia that also includes stressors specific for civilians. It lists 69 potential war stressors that relate to 9 categories of traumatic experience: active combat, witnessing of death or wounding, loss of organisational/military structure, war-related deprivation, injury, life in hostile surrounding, imprisonment/torture, combat exposure and civilian experiences specific for war in the Balkans. Participants were asked to mark the frequency of exposure which was recorded on the scale from 0 to 4 (1= did not experience, 1= once, 2= several times, 3=often, 4=almost every day). For the purpose of this study, the frequency of exposure is calculated as a dichotomous variable, 0= it never happened, 1= it happened at least once, with results ranging from 0 to 69.

To test the presence of early trauma, we applied the Early Trauma Inventory Self Report-Short Form—ETISR-SF (Bremner, Bolus, & Mayer, 2007). The ETISR-SF is a 27-item questionnaire, used for the assessment of physical, emotional and sexual abuse, as well as general traumatic experience that may have occurred before the age 18. Each of the items is answered “yes” (coded as 1) or “no” (coded as 0). There are additional three items at the end of the questionnaire. One of these asks the subjects to choose the one event that had the greatest impact on his or her life, and the other two items measure the subsequent reactions, that is, fear or depersonalisation.

### Procedure

Interviews and structured clinical interviews were performed by researchers/clinical psychologists with adequate educational background for the assessment and clinical experience with psychotraumatised individuals. All participants provided informed consent and fulfilled self-assessment questionnaires at their own home. The research was approved by the Ethical committee of the School of Medicine, University of Rijeka. Participants who completed the assessment received a compensation of €50.

### Statistical analysis

Descriptive statistics of predictors and criterion variables are presented as frequencies/percentages, or means and standard deviations for parametric measures. Descriptive statistics were used to report socio-demographic characteristics, the level of exposure to potentially traumatic events in youth, the number of potentially traumatic experiences during the war, and personality characteristics of the participants. Differences in exposure to early-life trauma and posttraumatic symptom severity between trauma and control group were tested using *t*-test for independent samples. Simple relations between potential predictors and severity of PTSD symptomatology are reported in the linear correlation matrix. Variables significantly correlated with PTSD were chosen for multiple regression analysis. Before the analysis, the data were tested for normality of distribution of independent and dependent variables. In addition, the variables were checked for univariate and multivariate outliers. Finally, potential predictors were tested for multicollinearity and singularity. Appropriate transformations were carried out where the data deviated from normal distribution. Multiple regression analyses were performed with the original and with transformed variables, and with and without the outliers. As both analyses yielded almost identical results, we decided to present the analysis of the non-transformed variables.

A multiple regression analysis was conducted to investigate the prediction of PTSD symptom severity using socio-demographic characteristics, traumatic experiences, and personality characteristics. To control the order of adding variables in the regression equation, the predictor variables were introduced in three steps. In the first step, only socio-demographic variables (age, education) were entered. Early traumatic events and exposure to war-related trauma were entered in the second step, and finally, personality traits from the five-factor model were added in the third step. For all tests, *p* values<0.05 were set as statistically significant.

## Results

### Trauma characteristics

The majority of the participants in the trauma group (*N*=293) experienced multiple war-related trauma with traumatic events ranging from 1 to 61 (*M*=11.48, SD=9.78). The most frequently experienced trauma come from the following categories: combat exposure, for example, *“*We were surprised by the enemy attack” (83.3%), civilian trauma experiences, for example, *“*We had to run away from our home” (79.6%), life in a hostile environment, for example, *“*For a long period I didn't know what happened to one or more of my family members” (76.6%), and war-related deprivation, for example, *“*I didn't sleep for more than 24 hours” (71.1%). Almost a half of the traumatised women witnessed death or wounding (46.4%). The least common events were imprisonment/torture (28.2%) and injury (5.9%) categories of war-related traumatic experiences (Table 1).

**Table 1:** Prevalence of war-related trauma

	Trauma group *N*=293
Traumatic event	*N* (%)
Active combat	44 (14.9)
Witnessing death or wounding	137 (46.4)
Loss of organisational structure	108 (36.5)
War-related deprivation	210 (71.1)
Injury	17 (5.9)
Life in hostile environment	226 (76.6)
Imprisonment/torture	83 (28.2)
Combat exposure	260 (88.3)
Civilian experiences	235 (79.6)

Of the 394 women included in the study, 270 (91.5%) from the trauma group and 92 (92.9%) from the control group experienced at least one potentially traumatic event under the age of 18 years. These potentially traumatic events included physical abuse, emotional abuse, sexual abuse, and general trauma (natural disaster, accident, death of an important other, etc.) (Table 2). The mean number of different early-life traumatic events was 5.70 (4.53) with the range 0–25 in the trauma group and 4.91 (4.02) with the range 0–18 in the control group. The difference between groups in the number of experienced early-life trauma is not statistically significant (*t*=1.67; *p*=0.095).

**Table 2:** Early-life trauma prevalence

Type of early-life trauma	Trauma group (*N*=293) *N* (%)	Control group (*N*=101) *N* (%)
General trauma	234 (79.3)	77 (76.2)
Physical abuse	240 (81.4)	83 (82.2)
Emotional abuse	132 (44.7)	45 (44.6)
Sexual abuse	60 (20.3)	19 (18.8)

The total number of traumas in the trauma group, which is the sum of early and war-related trauma, ranged from a small percentage who had experienced only one trauma (2%) to those with more than 40 (4.1%) different traumatic events, whereas the majority (more than 60%) experienced from 10 to 30 different types of trauma. The average number of summed traumatic event types was 17.18 (11.39).

According to CAPS, 61 (20.7%) women in the trauma group would be diagnosed with current PTSD, while none of the participants in the control group fulfil the criteria for current PTSD. Severity of PTSD symptoms (total CAPS score) in the trauma group (*M*=27.75, SD=24.71) is significantly higher (*t*=7.51; *p*=0.000) compared with the control group (M=8.63, SD=9.41).

### Relationship between trauma exposure, personality characteristics, and PTSD symptomatology

Table 3 lists the correlation coefficients between the predictor variables and symptom severity measured by the CAPS for the trauma group. The CAPS score is significantly correlated with all of the predictors except Agreeableness (Table 3).

**Table 3:** Correlations among socio-demographic variables, early-life trauma prevalence, exposure to war-related events, personality traits, and severity of posttraumatic stress disorder symptoms in trauma group

	Age	Education	Early trauma	War-related trauma	Neuroticism	Extraversion	Openness	Agreeableness	Conscientiousness
CAPS	0.34**	−0.22**	0.24**	0.47**	0.44**	−0.46**	−0.35**	0.02	−0.20**
Age		−0.35**	−0.03	0.21**	0.11	−0.34**	−0.37**	0.10	0.09
Education			−0.02	−0.16**	−0.15*	0.13*	0.31**	−0.05	0.04
Early trauma				0.16**	0.22**	−0.08	0.09	0.04	−0.16**
War-related trauma					0.23**	−0.25	−0.17**	−0.05	−0.12*
Neuroticism						−0.57**	−0.33**	−0.15*	−0.59**
Extraversion							0.65**	0.05	0.29**
Openness								0.08	0.16**
Agreeableness									0.27**
Conscientiousness									1

* *p*<0.01

** *p*<0.001.

A hierarchical multiple regression analysis was performed to assess the predictors of the level of PTSD symptoms, with the CAPS score for current PTSD symptoms as the dependent variable (Table 4). Model 1 containing socio-demographic variables accounted for 13.6% variance in total PTSD score (*R*^2^=0.136, *F*[2, 287]=22.58, *p*=0.000) with only age (*β*=0.321, *p* <0.001) being a significant predictor. Variables added in Model 2 (*R*^2^=0.313, *F*[2, 285]=36.75, *p*=0.000) contributed with additional 17.7% of variance. Both the number of early-life traumas (*β*=0.176, *p* <0.001) and the number of war-related traumas (*β*=0.364, *p* <0.001) were significant predictors. In the final model, personality traits were entered and they contributed with 14.5% of variance. The final model explained 45% of variance in PTSD symptoms (*R*^2^=0.458, *F*[2,284]=47.85, *p*<0.001) with age (*β*=0.164, *p*=0.002), number of early-life traumas (*β*=0.135, *p*=0.004), number of experienced war-related events (*β*=286, *p*=0.000), neuroticism (*β*=0.211, *p*=0.001), and extraversion (*β*=−0.175, *p*=0.013) having a significant unique contribution.

**Table 4:** Predictors of PTSD symptoms severity in trauma group

	Model 1: Socio-demographic variables		Model 2: Trauma characteristics		Model 3: Personality traits	
			
	*β*	*p*	*β*	*p*	*β*	*p*
Socio-demographic variables						
Age	0.321	0.000**	0.258	0.000**	0.164	0.002**
Education	−0.101	0.085	−0.064	0.223	−0.019	0.703
Trauma characteristics						
Early life trauma			0.176	0.000**	0.135	0.004**
War-related trauma			0.364	0.000**	0.286	0.000**
Personality traits						
Neuroticism					0.211	0.001**
Extraversion					−0.175	0.013*
Openness					−0.110	0.085
Agreeableness					0.045	0.331
Conscientiousness					−0.012	0.833

* p<0.05

** p<0.01

## Discussion

The results of the presented study show a high prevalence of posttraumatic symptoms in the cohort sample of women 10 years after the war in Croatia. In addition to exposure to the war-related traumatic events, it shows the importance of the impact of traumatic experiences in childhood and/or early adolescence as well as personality traits in the explanation of posttraumatic symptom severity in female war victims.

More than 10 years after the war in Croatia, the level of posttraumatic stress symptoms in women exposed to war stressors is relatively high. Taking the CAPS cut-off score of 65, more than 20% of participants have a sufficient level of symptoms that could be diagnosed with current PTSD. This prevalence rate is consistent with other studies on civilian victims after wars (Letica Crepulja, Salcioglu, Frančišković, & Basoglu, 2011; Schick, Morina, Klaghofer, Schnyder, & Müller, 2013), and it is significantly higher than the average prevalence rates found in populations not living in post-conflict areas (Breslau et al., 1999; Kessler et al., 1995). A large majority of the participants in the study had been exposed to at least one war-related traumatic event, with the average of 11 different war-related events. These results are in line with other studies on civilians, done in similar settings, using similar methodology (Farhood & Dimassi, 2012). Prevalence rates of early-life traumatic stressors are very high in this sample. In our sample, over 90% of the participants in both trauma and control groups reported at least one potentially traumatic event before the age of 18. Groups did not differ in the average number of early-life trauma which suggests that the level of pre-war traumatisation was equal for those who later experienced war-related trauma as for those that were not exposed to war-related activities. However, severity of the post-war PTSD symptoms was significantly higher in the war-traumatised group of women suggesting that the observed symptoms resulted from the cumulative effect of early-life and war-related trauma and are not just simple continuation of posttraumatic problems from early-life traumatisation.

A regression model that included age, early-life trauma, war-related trauma, neuroticism, and extraversion as statistically significant predictors explained 45.8% of variance of PTSD symptoms registered by the CAPS in the trauma group. Consistent with the literature, the number of war-related traumatic events explained the largest proportion of the variance in PTSD severity. Levels of posttraumatic symptomatology were higher for women experiencing more traumatic events related to the war. These results are in support of the well-established dose–response relationship between the frequency of exposure to a variety of traumatic war-related events and the severity of posttraumatic symptoms (Ogle et al., 2014; Bresslau et al., 1999). A rather important result of this study is the similar cumulative effect in the early-life trauma in relation to PTSD. Greater exposure to traumatic events in childhood and early adulthood is uniquely associated with higher levels of PTSD symptoms registered after war traumatisation. These results are in favour of the hypothesis that previous exposure to traumatic events is associated with higher risk for PTSD after a subsequent trauma and are consistent with the results of several studies, that is, Cloitre et al. (2009) and Owens et al. (2009). It seems that the trauma experienced in young age is associated with greater vulnerability for PTSD later in life; it contributes to the chronification of the symptoms and may influence responses to the future trauma. Possible explanation of those associations could be that exposure to maltreatment in young age leads to a specific vulnerability in which learned helplessness, low self-esteem, and development of maladaptive coping mechanisms influence the responses to the future trauma (Widom, 1999). Alternative explanations of the relation between early-age trauma and post-war PTSD, as suggested by some research, could include common risk factors for exposure to traumatic events and development of PTSD (Cougle, Resnick, & Kilpatrick, 2009; Lanius, Frewen, Vermetten, & Yehuda, 2010).

After adding personality traits in the regression model, only neuroticism and extroversion stood out as statistically significant predictors of posttraumatic symptom severity. Controlled for age and the level of traumatisation, women with higher neuroticism and lower extraversion tend to have more PTSD symptoms. This is somewhat in line with previous studies suggesting a distinct personality profile for those who suffer from PTSD consisting of higher neuroticism, lower extraversion, and lower agreeableness (Löckenhoff et al., 2009). Our study did not find significant relations of other personality traits with PTSD symptoms. A positive relationship between PTSD symptoms and neuroticism has been well explained (Jakšić et al., 2012). Neuroticism and PTSD share many features such as vulnerability to experience anxiety and general distress, and tendency to avoidance. Those higher in neuroticism are more easily upset and distressed, which well reflects PTSD. There are a number of potential mechanisms through which neuroticism may be related to the risk for PTSD. For example, high levels of neuroticism are often associated with a tendency to utilise less social support, endorse a higher level of threat appraisal and negative affect, and have a greater utilisation of emotion-focused coping styles (i.e., appraisals of high situational demand relative to coping resources) (Borja, Callahan, & Rambo, 2009; DeLongis & Holtzman, 2005; Erbes, Curry, & Leskela, 2009). Each of these factors is associated with poorer mental health outcomes (Folkman, Lazarus, Dunkel-Schetter, DeLongis, & Gruen, 1986; Penley & Tomaka, 2002). Thus, neuroticism may exacerbate the association between the severity of traumatic events and the development of PTSD through these pathways. Extraversion, on the other hand, has been linked to PTSD resilience (Campbell-Sills, Cohan, & Stein, 2006; Fauerbach, Lawrence, Schmidt, Munster, & Costa, 2000; Vrana, 2001). Higher extraversion is associated with greater social support, a higher level of challenge appraisals (i.e., appraisals of greater coping resources relative to situational demand), positive affect, higher self-efficacy, and greater utilisation of problem-focused coping styles. Each of these mechanisms may independently buffer against psychological distress. More specifically, extraversion may influence one's PTSD risk through the tendency to prefer prosocial activities and to demonstrate high warmth, which may lead one to both seek out and more successfully utilise social support. Also, someone high in extraversion may be more deliberate in utilisation of support and use problem-focused coping styles for achievement striving purposes, and thus reduce their risk for PTSD.

### Strengths and limitations

This study adds to the field of posttraumatic stress research in populations of women in several aspects. Previous studies that examined relations of childhood trauma, war-related trauma, personality, and posttraumatic symptoms rarely included civilian women in their samples. To our best knowledge, this is one of the few researches that examine the prediction models of posttraumatic symptom severity in a sample of female civilians who were exposed to war-related trauma. Structured clinical interviews and validated self-reporting instruments allowed us to obtain reliable data. Snowballing method allowed us to find cases that otherwise would not report themselves due to the sensitive and rather private issue of our research.

This research also has several limitations. The study design is retrospective and all the data about war-related traumatisation were collected approximately 10 years after the war. Such a long period increases the likelihood of recall bias and consequently can lead to potentially unreliable data on trauma, that is, under-reporting or over-reporting of traumatic events. Given that participating in this study could not result in any personal gain or privilege, the likelihood of deliberate over-reporting is small. On the other hand, avoidance of traumatic and painful recollections could also lead to under-reporting of certain traumatic events. Another major limitation to the study is the lack of examination of post-war civilian traumatic experiences. It is possible that the results to some extent reflect the exacerbation of PTSD symptoms due to post-war civilian trauma. Because of the snowball sampling technique, the representativeness of the sample and consequently the possibility of generalisation of the results are limited. In addition, 89 participants refused to participate due to lack of time or unwillingness to recall war-related events which might have an impact on results.

## Conclusions

The results showed that age, level of education, traumatic experiences in childhood and early adolescence, exposure to war-related traumatic events, and personality traits from the five-factor model explained 45.8% variations of the PTSD symptom severity in war-traumatised women. Older age, exposure to early trauma, exposure to war-related traumatic events, high neuroticism, and low extraversion were found to be independent predictors. In order to shed more light on the relations of early traumatic experiences and post-war PTSD symptomatology, it would be useful to define more precisely and examine the independent role of each early traumatic experience and possible mediating effects. Recommendations for future research could include methodologically more rigorous sampling methods, which would enable higher representativeness of the results.

The presented results, along with similar studies, suggest clinical implications in dealing with war-traumatised females. Interventions aimed at the effects of war-related trauma in women could likely be improved with a focus on both pre-war traumatisation and post-conflict factors. Potential vulnerability factors or triggers of posttraumatic symptoms may be related to the specific experiences of early-life trauma. A better understanding of interaction of these factors could allow the creation of customised therapeutic interventions aimed precisely at consequences of particular experiences. Furthermore, knowledge about certain risk factors in terms of personality characteristics might be helpful in designing services of preventive care and treatment.
